# Industrial Robot Control by Means of Gestures and Voice Commands in Off-Line and On-Line Mode

**DOI:** 10.3390/s20216358

**Published:** 2020-11-07

**Authors:** Wojciech Kaczmarek, Jarosław Panasiuk, Szymon Borys, Patryk Banach

**Affiliations:** Faculty of Mechatronics, Armament and Aerospace, Military University of Technology, Kaliskiego 2 Street, 00-908 Warsaw, Poland; wojciech.kaczmarek@wat.edu.pl (W.K.); jaroslaw.panasiuk@wat.edu.pl (J.P.); patryk.banach95@gmail.com (P.B.)

**Keywords:** Kinect, industrial robot, vision system, RobotStudio, Visual Studio, gesture control, voice control

## Abstract

The paper presents the possibility of using the Kinect v2 module to control an industrial robot by means of gestures and voice commands. It describes the elements of creating software for off-line and on-line robot control. The application for the Kinect module was developed in the C# language in the Visual Studio environment, while the industrial robot control program was developed in the RAPID language in the RobotStudio environment. The development of a two-threaded application in the RAPID language allowed separating two independent tasks for the IRB120 robot. The main task of the robot is performed in Thread No. 1 (responsible for movement). Simultaneously, Thread No. 2 ensures continuous communication with the Kinect system and provides information about the gesture and voice commands in real time without any interference in Thread No. 1. The applied solution allows the robot to work in industrial conditions without the negative impact of the communication task on the time of the robot’s work cycles. Thanks to the development of a digital twin of the real robot station, tests of proper application functioning in off-line mode (without using a real robot) were conducted. The obtained results were verified on-line (on the real test station). Tests of the correctness of gesture recognition were carried out, and the robot recognized all programmed gestures. Another test carried out was the recognition and execution of voice commands. A difference in the time of task completion between the actual and virtual station was noticed; the average difference was 0.67 s. The last test carried out was to examine the impact of interference on the recognition of voice commands. With a 10 dB difference between the command and noise, the recognition of voice commands was equal to 91.43%. The developed computer programs have a modular structure, which enables easy adaptation to process requirements.

## 1. Introduction

The development of electronics, especially sensorics, results in a constant change in the way people interact with electronic devices. An example of using gestures and voice commands to operate devices can today be TVs, computers, building automation systems, mobile robots, etc. The development of electronics and vision systems contributes to the development of a new way of human interaction with devices, which creates completely new possibilities for designing and applying new computer applications. This is particularly evident in interfaces to video surveillance and game applications. A major advantage of using gestures and speech in interaction with the machine is the possibility of dynamic adaptation. The movements of the human body can be considered as segments [1] that express a specific meaning in specific time periods. Hence, during a conversation, people often gesture, emphasizing the meaning of spoken words. Therefore, sequences of gestures can indicate to the machine the next actions to be performed. By adding voice commands to this, it turns out that the method of communication with machines will become very close to the method used in everyday interpersonal relations. When considering this problem, taking into account robotics, it should be noted that the use of gestures and voice commands will make it easier to program and operate very complex devices even for beginners.

Already today, Industry 4.0 integrates people and digitally controlled machines with the Internet and information technologies in various fields of technology [2,3]. On the one hand, the information flow is carried out at the level of the production process between the machines; on the other hand, the process information obtained supports company management systems, which makes it easier to forecast production efficiency.

During the implementation of industrial processes, it is necessary to take into account many technological and hardware parameters [4,5,6,7]. A great challenge is to achieve high productivity and product quality in relation to the costs incurred for the construction of production lines and production itself. Therefore, it is necessary to develop, as much as possible, a flexible production system that would ensure that orders are met today, and in the future, this would allow adapting to changing customer needs. Techniques and tools are sought to analyse the current technological processes with the possibility of anticipating future use of the system [8,9].

Research efforts are being carried out worldwide to highlight the importance of human-machine integration in various aspects. An example is the study [10], which highlighted the importance of human-machine integration in the context of obtaining data for reliability analysis.

In the near future, vision systems used for many years in industrial applications may be used for programming industrial robots or may give the possibility of easier cooperation between the operator and machines at the workstation [11]. Traditional robot programming with the use of dedicated teaching panels slowly becomes a rarity and is replaced by computer programming [12,13,14]. Thus, the question will arise as to what form the programming of robots will take in the future and whether it is possible to create flexible programming that will allow the operator to interact with the robots using natural tools available to man, such as body movement (gestures) or voice. In research laboratories, applications are created that bring us closer to this goal. Developments on programming and controlling robots using gestures and voice are carried out in many fields, e.g., industrial applications (assembly robots), transport (mobile robots), and home use (cleaning robots) [15]. An example can be projects in which images are used to recognize hand gestures in order to control a mobile robot through a specially made control circuit [16]. Of course, the engineer’s or operator’s safety is important in this case, but modern robots today are equipped with safety systems that allow for full collaboration between robots and people [17].

The development and testing of industrial robot control applications can take place in virtual reality in off-line mode. Robot manufacturers offer environments for programming and modelling robots (e.g., RobotStudio by ABB, ROBOGUIDE by FANUC, RT ToolBox by Mitsubishi Electric, KUKA SimPro by KUKA). The modern approach allows creating a virtual controller, which is a digital twin of its real counterpart. This means that it has exactly the same functionality and that the way and principle of its operation almost correspond 100% to the operation of the real device [18]. This gives great possibilities especially because of the functional analysis of software under development. Many researchers use the Kinect sensor for gesture control of robots [19,20,21,22,23,24,25]. An important part of robot programming is preparing an optimal trajectory and gripping points [26,27,28,29,30,31]. An interesting use of the Kinect sensor is its use as a 3D scanning sensor mounted on a FANUC R2000 industrial robot [32]. This approach allows the scanning of large objects on the one hand, but on the other, it is flexible and easily reprogrammable. 3D scanners can also be used to scan larger areas, such as robotic production cells or even entire buildings [33,34]. The limitations of the sensor’s design must be taken into account, as the use of the IR detector requires appropriate environmental conditions to eliminate interference. This solution solves many important problems, but most of it is limited to control by gestures only, without considering the possibility to control voice commands as well. Moreover, it seems to be advisable to implement solutions allowing creating digital stations and using virtual environments.

This paper presents a method of controlling an industrial robot with the use of gestures and voice commands. The solution of the problem required the development of a computer program in the C# language in the Visual Studio environment, which allows obtaining data from the Kinect V2 sensor and is responsible for communication with the application developed for the industrial robot [35]. During the development of a program in Visual Studio, the ABB.Robotics.Controllers library was used to connect directly to the robot controller, set the robot’s operating parameters, and run the robot control program. The Microsoft.Kinect library was used to detect, connect to the application, and receive and interpret data from Kinect V2. This library has the types of variables and mechanisms to operate this device. Voice control of the robot is provided by the System.Speech.Recognition library. The TCP/IP protocol and the System.Net library are responsible for proper communication and smooth robot control. The user interface is based on a WPF (Windows Presentation Foundation) application.

The application for the IRB 120 robot from ABB company was developed in the RAPID programming language. It was decided that the application would be two-threaded. The main thread is responsible for the execution of the robot’s movements and logic related to the production task. The second thread, working in the background of the main thread, is responsible for communication via Ethernet with the application supporting the Kinect sensor.

The prepared software enables testing of the application (e.g., communication of devices and realization of production tasks) and the simulation of the robot’s work in off-line mode with the use of the digital twin and on-line control of the real robot. Tests of the real robot allowed validating the correctness of digital twin’s operation.

Section 2 contains a project of the robotic test station with its digital twin. Section 3 presents a description and the principle of operation of the control programs and the results of the tests carried out. Section 4 summarizes the results of our research.

## 2. Station Design

**Figure 1:**
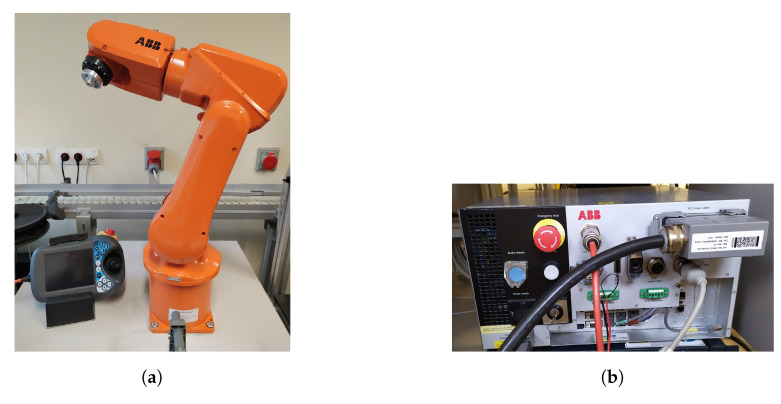
General view: (**a**) ABB IRB 120 robot with FlexPendant; (**b**) IRC5 Compact robot controller.

**Figure 2:**
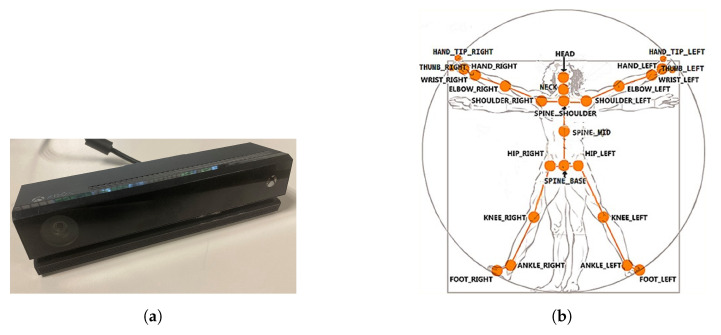
Kinect V2: (**a**) general view; (**b**) recognizable body points.

Tests were carried out on a research station in the Robotics Laboratory of the Military University of Technology. The station consists of:

- The ABB six-axis IRB 120 robot with the IRC5 Compact controller (Figure 1),
- The Kinect V2 sensor (Figure 2),
- A PC.

### 2.1. ABB IRB 120 Robot with the IRC5 Compact Controller

The IRB 120 is one of ABB’s smallest industrial robots. It weighs 25 kg and can handle loads up to 3 kg with a range of 580 mm [36].

### 2.2. Kinect V2 Sensor

The Kinect V2 sensor consists of three components, an RGB camera, a depth sensor (emitter and infrared camera), and a set of four microphones [37], as shown in Figure 2, which give it the functionality to control it with gestures and speech.

A depth sensor is used to track a person’s movement. The infrared emitter sends an infrared beam, which is distorted upon impact with objects or people in its path and is recorded by the infrared camera. From the read data, a room image is created, and the user or users are identified. A set of microphones is used to collect voice information that can be used as control commands.

The Kinect V2 “sees” a person as a set of 25 points (Figure 2b). Their interpretation allows tracking and reading the movements of a person or group of up to six people. Thanks to the points, it is possible to assess in which position a person is currently in front of the device. From these coordinates, it is possible to separate the required coordinates $\left(X_{n-1},X_{n},Y_{n-1},Y_{n}\right)$, which correspond to the right and left hand. Vector Equations (1)–(3) can be used to convert these coordinates to joint angles [21].

(1) $$A=X_{n}-X_{n-1}$$

(2) $$B=Y_{n}-Y_{n-1}$$

(3) θ=ArcTan(A,B)

where θ is the angle between two adjacent joints, $X_{n-1},X_{n}$ the X coordinates of the joints, and $Y_{n-1},Y_{n}$ the Y coordinates of the joints.

### 2.3. PC

A computer with Windows 10 was used to control an industrial robot with gestures and voice commands. Thanks to the developed control programs, the computer provided support for the Kinect sensor (Visual Studio) and communication with the robot controller (RobotStudio). Kinect for Windows SDK 2.0 (Microsoft.Kinect library) was used to operate the Kinect sensor, while PC SDK 6.07 (ABB.Robotics.Controllers) libraries were used to operate the robot controller.

The key issue for this project was the detection of human body points using the Microsoft Kinect sensor and the use of microphones. A full image processing logic was developed to control the industrial robot using gestures. In addition, a set of microphones was used to control the robot using voice commands.

### 2.4. Test Station and Its Digital Twin

**Figure 3:**
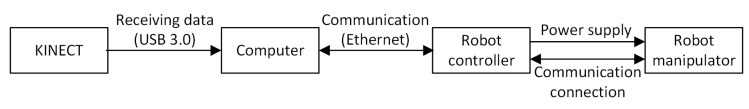
Flowchart of the station.

The flowchart of the station is shown in Figure 3.

**Figure 4:**
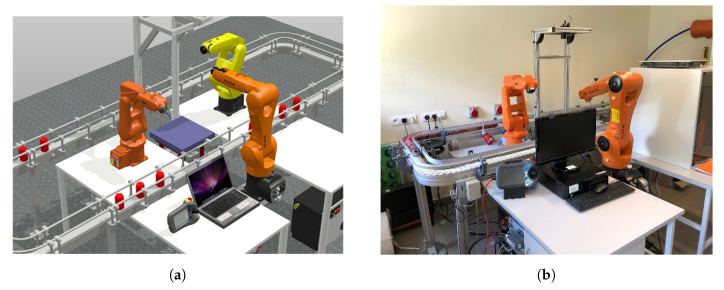
General view of the robotised station: (**a**) virtual; (**b**) real.

One of our aims was to develop the digital twin of the real robotic position (Figure 4). This enabled the creation, modification, and testing of software in a safe way, without the need to work on-line with the real robot.

The backup of the real robot was used to create a digital twin system. In the RobotStudio environment, and the backup generated a robot system that runs on a virtual controller. Such an approach allowed reproducing the real robot controller with almost 100% accuracy. This means that the robot model in a virtual environment has the functionality and features of a real device. This enables the robot model to be programmed taking into account all the capabilities and limitations of the real robot, and the results obtained during the simulation were reliable.

## 3. Application Design

**Figure 5:**
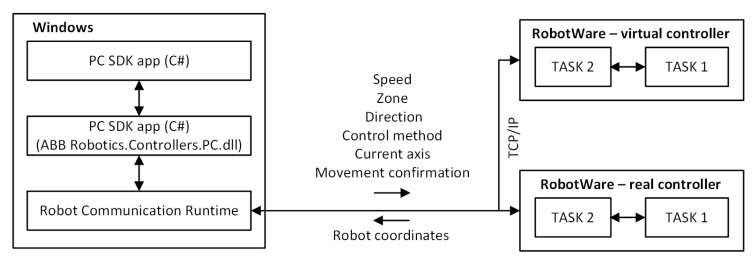
General diagram of communication between the application and the robot controller.

The integration of devices included in the station (IRB 120 robot, Kinect V2 sensor, computer) required the development of applications enabling the operation of the Kinect sensor and the implementation of an industrial robot control process, as well as ensuring communication between devices (Figure 5).

A control application in the C# language was developed, which was responsible for image processing and communication among the Kinect controller, computer, and robot controller [38,39]. The application was also equipped with a graphical user interface, allowing for free operation without the need to interfere with the program code.

The Microsoft.Kinect library was used to operate the sensor, which allows detecting and connecting to the connected device [40]. For the purposes of the application, an object representing the Kinect was prepared, which made it possible to connect the device with the application being developed. In the next step, an object representing a human being as a whole was declared, and the created logic and software functions allowed to searching for objects representing elements of the human body in the data sent by the Kinect sensor. Next, the authors’ application divided the obtained object into components representing characteristic points of the body, e.g., elbows, shoulders, head, and the state of the hand (open, closed). After recognizing the body of the operator, the position of individual points in relation to each other (Figure 2b) was calculated. The operator’s skeleton is displayed on the user interface as a mirror image to facilitate control, and it was done by developing a sub-program using the points received from the Kinect. The application scaled the received image and displayed it as characteristic points between which lines were drawn, creating a skeleton representing the operator. Based on the operator object received from the Kinect, gestures were proposed to give commands to the robot. Depending on the position of the arms and palms, the developed application allows performing the set of tasks according to the work algorithm.

To implement voice control, the Kinect V2 is connected to a computer and set in Windows 10 as the default microphone (another external microphone can be used). The software was developed using the System.Speech.Recognition library [41]. In the first step, a voice command recognition engine was developed, and the words were declared as text, which were then converted into a dictionary. The prepared dictionary was uploaded to the speech recognition engine, which allows the detection of key words as operator voice commands. On the basis of the recognized commands, a part of the application in which the commands are assigned to the signals controlling the robot was developed.

The application developed in the Visual Studio environment communicates with the robot via Ethernet using the TCP/IP data transmission protocol (Figure 5). The ABB.Robotics.Controllers and System.Net libraries were used to create the application [42]. This application uses ABB libraries to send data to the Robot Communication Run Time module, which is responsible for code synchronization and data transfer in both directions. The authors assumed that the application would allow connecting to all robot controllers in the Ethernet network. In order to make this possible, a search engine was developed for the controllers available in the network. The class implemented in the code allows connecting to the selected controller. The developed application allows direct access to the robot controller before starting the control program, setting the data values and selected parameters of the robot, and uploading them to the controller, e.g., start points of the robot program, manipulator speed, and accuracy. It is also possible to start or stop the robot control program. The next routine allows influencing the robot’s operation also while it is performing its program. This gives the possibility of sending and receiving signals, e.g., sending control signals and receiving the current position of the robot’s manipulator.

The control program for IRB 120 was developed in the RAPID language in the RobotStudio environment. The program is a two-threaded application in which:

- Thread 1 (Main) is responsible for setting the parameters, interpreting the robot’s direction of motion, and controlling the robot’s movements.
- Thread 2 is responsible for the TCP/IP communication between the robot and the computer and transferring the data received from the Kinect sensor to the main thread.

This made it possible to make the robot’s motions independent of the actions responsible for data exchange. The threads operate asynchronously based on the same global data (persistent data (PERS)). Thus, if the value of data in one thread changes, it is automatically updated in the other thread. This is of key importance in the control process, because this solution allows controlling an industrial robot using gestures and voice commands without visible delays.

### 3.1. User Interface

In addition to the full functionality of this application, which allows for controlling the industrial robot by means of gestures and voice commands, an intuitive graphical interface was developed, which allows for easy startup and control of the station.

**Figure 6:**
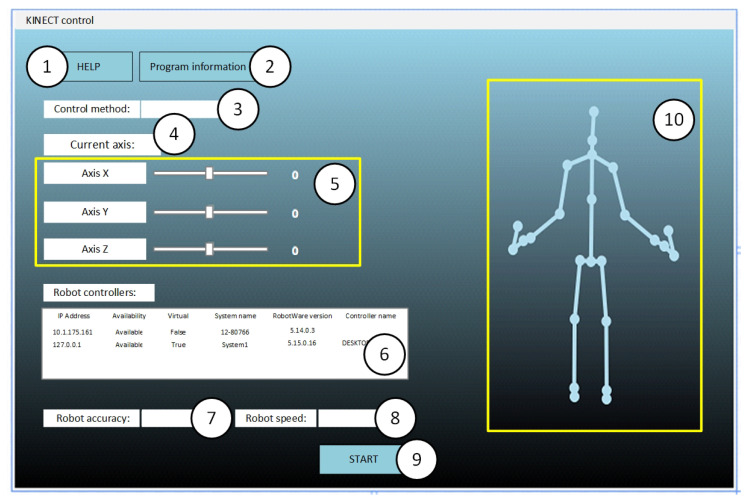
Graphical interface of the developed application: 1—help button, 2—program information button, 3—control method list, 4—current controlled axis, 5—current *x*, *y*, *z* axis position field, 6—list of available robots, 7—robot accuracy window, 8—robot speed selection window, 9—start button, 10—operator’s “skeleton” field.

The user interface was developed in Microsoft Visual Studio 2017 in the C# language. This application enables the user to control an industrial robot by hand movement in 3D space (hand tracking and gestures) (Figure 6) and to control the robot by voice commands.

### 3.2. Application Tests

The startup tests of the application were performed using the digital twin in the RobotStudio environment (Figure 4a). The developed digital twin allowed for:

- launching all prepared applications on one computer. The virtual station model was launched in the RobotStudio environment, and communication with the application supporting the Kinect sensor and with the user interface was carried out using the localhost;
- launching the prepared applications on two computers. The virtual station model was launched in the RobotStudio environment on one computer(simulation of the operation of a real station). The application supporting the Kinect sensor and the user interface was launched on the second computer. The computers were connected to the local Ethernet.

**Figure 7:**
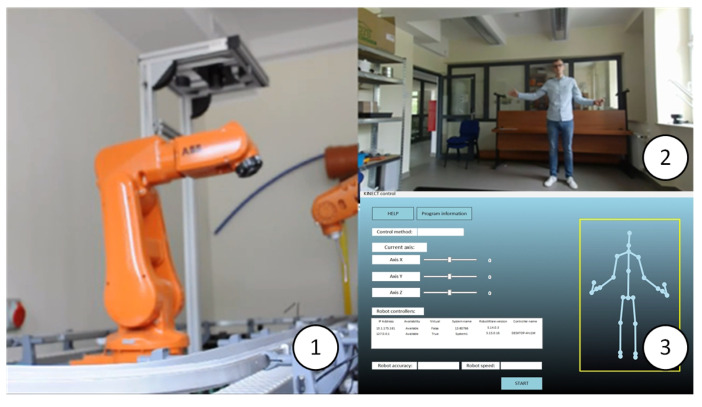
Views of the test station: 1—ABB IRB 120 robot, 2—operator, 3—operator interface window.

During the tests, the correctness of the robot’s task performance was verified both on the basis of performed gestures and voice commands. The adopted solution allowed the execution of the tests. Validation of the application’s correctness was carried out on the real station (Figure 4b and Figure 7).

## 4. Results and Discussion

A number of tests were carried out to verify the correctness of the robot’s reaction to gestures and voice commands. Tests were conducted using a digital twin and a real robot. Both single movements and complex tasks (e.g., pick and place) were tested.

In the case of robot control by means of gestures, tests were carried out on the performance of commands by the robot in the form of motion in selected directions (Table 1), following the operator’s hand (Figure 7), performing a complex task, and controlling the robot’s input signals. No communication errors or unrecognised commands were observed. The dynamics of the Kinect sensor and, above all, the acceleration and speed capability of the robot are limited when the robot follows the hand movement. This type of control seems to be useful when tasks need to be carried out remotely (in a place that is difficult to reach or far away), and the time of task completion does not play a significant role here. Gestures seems to be a more promising control method than hand tracking, because it may execute complex tasks for the robot (e.g., assembly, sorting). The main advantage in this case is that the operator can start the robot, indicate the task, or turn off the robot without having to use an additional control panel.

Tests of the delay of the robot’s reaction to voice commands were conducted. Selected test results are presented below (Table 2 and Table 3).

**Figure 8:**
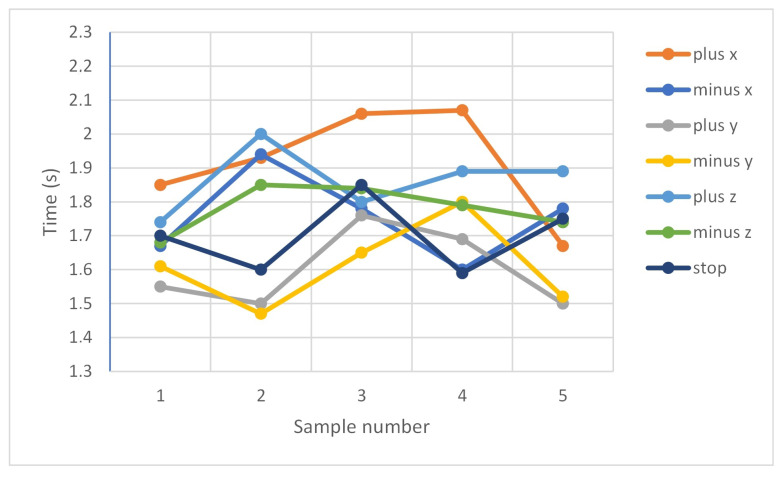
Measurement of the delay of the robot’s reaction to the voice command: real station.

Figure 8 shows the delay of the robot’s reaction to the voice commands on the real station.

**Figure 9:**
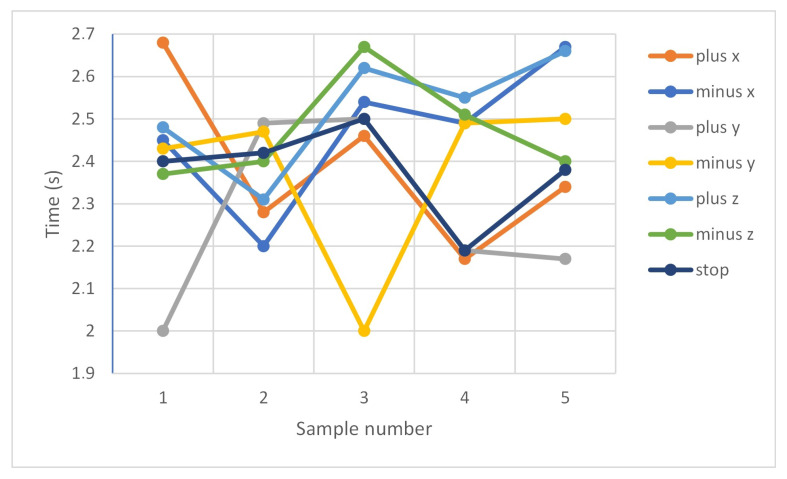
Measurement of the delay of the robot’s reaction to the voice command: virtual station.

Figure 9 shows the delays expressed in seconds for voice control commands on the virtual station.

Table 2 and Table 3 indicate a slower robot response in the RobotStudio environment. There is a difference of 0.92 s between the fastest average response of the real robot as compared to a virtual robot. In both cases, the robot best responds to commands to control along the y-axis. The average response rate for the actual device is 1.74 s and for the virtual one 2.41 s. This may be due to the fact that Robot Studio is not working in real time and the insufficient computing capacity of the computer used was affecting the processing time. Delay may also be caused by the fact that communication was performed using a TCP/IP over a network and that the robot controller may have tasks of higher priority to perform first.

The next study evaluates the influence of sound interference intensity (20 dB, 40 dB, 60 dB) on the ability to control the robot by voice commands. The “Sound Master” application was used to measure the interference. The measuring device was located next to the Kinect sensor. A music track without vocals was used as a disturbance. The commands for the robot were given in a calm voice of 70 dB in intensity. A number of tests were carried out on a virtual and real station, changing the robot’s parameters. For 20 dB and 40 dB interference, there were no problems with the performance of commands. The tables below show the results for 60 dB motion commands.

The results presented in Table 4 show that commands of 70 dB in intensity can be incomprehensible for a robot system equipped with the Kinect sensor if the interference is at 60 dB. The achieved recognition rate of voice commands is equal to 91.43%. This value might be enough for performing laboratory tests, but is definitely not acceptable in industrial environments. However, it should be noted that in the case of simple commands (for the virtual and real system), the “plus y” command caused the problem, while other commands were recognized correctly. Therefore, it is important that the selected commands are easily and clearly identified by the system, especially in industrial conditions (with noise).

## 5. Conclusions

Human interaction with an industrial robot using a vision system equipped with microphones seems to be an interesting and promising solution for future robot programming. Teaching the trajectory of industrial robots with gestures and voice commands may prove to be one of the methods of robot programming in the future. The combination of gestures and voice commands also seems to be an interesting issue in terms of robot control, especially since the advantage of using gestures and voice commands in interaction with the machine is the ability to adapt dynamically. For humans, this form of communication seems to be the most intuitive, as we learn from childhood to use gestures for more accurate expression of our feelings, emotions, and needs. In our lives (in direct contacts), speech and gestures coexist, and therefore, they should also be combined in interactions with machines. As recent trends indicate, in the near future, this type of control will become a standard in cars, where the leading manufacturers already offer such functionality. An example is the handling of navigation and multimedia systems. Control of this type has many supporters, although there are also opinions that voice and gesture control systems do not work precisely and unambiguously enough. This is mainly due to the appearance of interferences (e.g., noise or sounds of music in a moving car) and inefficient algorithms of voice and gesture analysis and processing. Currently developed algorithms are getting better and better, which allows us to think that they will also be used in factories. Expanding the systems of industrial robots and their control programs with the possibility to control by means of gestures and voice commands will make it easier for operators of production stations to communicate with machines and will make them able to operate extended stations after a short training. It should be expected that in the future, this type of control will especially find its application in areas where humans will cooperate with a robot (e.g., collaborating robots (cobots)). The possibility of easier communication with cobots (without additional distractions for operators) will allow for closer and faster cooperation, similar to the level of cooperation between cooperating people.

This paper focuses on the presentation of prepared software, which allows controlling an industrial robot by means of gestures and voice commands. ABB IRB 120 robot was used as an example, but the developed software can be easily integrated with other robots. For the IRB 120 robot, we developed a multithreaded control program that allows for a flexible tasks realization. Our aim was to develop an inexpensive and universal solution. Tests showed that gesture control allows for closer cooperation between man and machine. Additional voice commands extend the possibilities of controlling the robot without the need to focus all the operator’s attention on it. The presented test results prove that the recognition of voice commands depends on the noise level. It was noted that the 10 dB difference between command and noise provided 91.43% recognition of the commands.

The paper also presents the possibility of performing tests based on digital twins. Already today, such an approach allows creating control programs for robots and conducting analyses of the work of robotic stations, even entire production lines and factories. The simulation results are particularly important for integrators (at the start of production) and managers (who manage production and conduct analyses of market changes). The research carried out, using a real robotic station and a virtual robotic system, shows that in the case of voice handling, the average difference between real and virtual robot performance was 0.67 s. This difference may result from the fact that part of the API executes on a non-real-time operating system, and the communication is done using a TCP/IP over a network. Furthermore, the robot controller sometimes has tasks of higher priority to perform first. Such analyses allow for a quick response to market changes and the introduction of changes in production. The use of digital twins allows preparing and testing the introduced changes without stopping production.

In the next stage of research, the industrial robot will be controlled by means of gestures and voice commands taking into account the robot’s safety zones. Moreover, with the use of digital twins, tests of controlling the robot by means of gestures and voice commands in virtual reality with HTC VIVE Cosmos goggles will be conducted.

## Figures and Tables

**Table 1:** Gestures used in robot control.

Gesture *	Robot Action	Operator’s Body Position
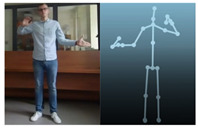	Axis X move forward	Right hand on the left, left hand below shoulder
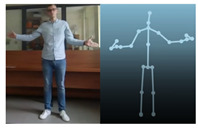	Axis X move backwards	Right hand on the right, left hand below shoulder
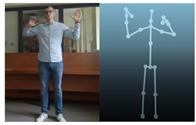	Axis Y move forward	Right hand on the left, left hand between shoulder and head
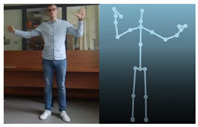	Axis Y move backwards	Right hand on the right, left hand between shoulder and head
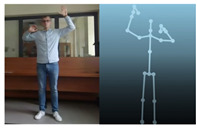	Axis Z move forward	Right hand on the left, left hand above head
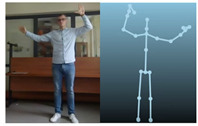	Axis Z move backwards	Right hand on the right, left hand above head
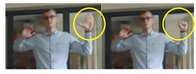	Confirm movement	Close left hand

* The skeleton field is mirrored.

**Table 2:** Measurement of the delay of the robot’s reaction to the voice command: real station.

Sample Nb	Commands						
	**“plus x”**	**“minus x”**	**“plus y”**	**“minus y”**	**“plus z”**	**“minus z”**	“**stop**”
Sample 1 (s)	1.85	1.67	1.55	1.61	1.74	1.68	1.70
Sample 2 (s)	1.93	1.94	1.50	1.47	2.00	1.85	1.60
Sample 3 (s)	2.06	1.78	1.76	1.65	1.80	1.84	1.85
Sample 4 (s)	2.07	1.60	1.69	1.80	1.89	1.79	1.59
Sample 5 (s)	1.67	1.78	1.50	1.52	1.89	1.74	1.75
Average delay (s)	1.92	1.75	1.60	1.61	1.86	1.78	1.70

**Table 3:** Measurement of the delay of the robot’s reaction to the voice command: virtual station.

Sample Nb	Commands						
	**“plus x”**	**“minus x”**	**“plus y”**	**“minus y”**	**“plus z”**	**“minus z”**	**“stop”**
Sample 1 (s)	2.68	2.45	2.00	2.43	2.48	2.37	2.40
Sample 2 (s)	2.28	2.20	2.49	2.47	2.31	2.40	2.42
Sample 3 (s)	2.46	2.54	2.50	2.00	2.62	2.67	2.50
Sample 4 (s)	2.17	2.49	2.19	2.49	2.55	2.51	2.19
Sample 5 (s)	2.34	2.67	2.17	2.50	2.66	2.40	2.38
Average delay (s)	2.39	2.47	2.27	2.38	2.52	2.47	2.39

**Table 4:** Effects of 60 dB interference on the robot’s operation.

Sample Nb	Commands						
	**“plus x”**	**“minus x”**	**“plus y”**	**“minus y”**	**“plus z”**	**“minus z”**	**“stop”**
Sample 1	ok	ok	ok	ok	ok	ok	ok
Sample 2	ok	ok	ok	ok	ok	ok	ok
Sample 3	ok	ok	ok	ok	ok	ok	ok
Sample 4	ok	ok	x	ok	ok	ok	ok
Sample 5	ok	ok	x	ok	ok	ok	ok
Sample 6	ok	ok	x	ok	ok	ok	ok
Sample 7	ok	ok	x	ok	ok	ok	ok
Sample 8	ok	ok	ok	ok	x	ok	ok
Sample 9	ok	ok	ok	ok	ok	ok	ok
Sample 10	ok	ok	x	ok	ok	ok	ok
Recognition rate (%)	100	100	50	100	90	100	100
